# Student learning about UK health services in a cross-border curriculum partnership; the London-Cyprus experience

**DOI:** 10.15694/mep.2020.000058.1

**Published:** 2020-03-30

**Authors:** Shehla Baig, Stella A. Nicolaou, Denise Lawrence, Janette Myers, Mursheda Begum

**Affiliations:** 1 St. George’s University of London; 2 University of Nicosia; 3 King’s College London

**Keywords:** Crossborder curriculum partnerships, International medical education, Problem-based learning, Health Services and education

## Abstract

This article was migrated. The article was marked as recommended.

Purpose of the article

Cross border partnerships require curricula, faculty and students to negotiate challenges associated with national regulatory frameworks, contexts and cultures. This study investigated student attitude and behaviours when encountering learning about health services in host and home students in the context of problem based learning.

Materials and Methods

First year graduate entry students’ health systems interest and exposure and their perceptions of the dynamics of learning in PBL were investigated via a questionnaire comprising open and closed questions.

Results and Conclusions

Results showed a difference between home and host students in the ways they learned about home health systems and their attitudes to the value of learning about home and international health systems. There was no difference in the quantity of health service related learning objectives generated. Both groups reported noticing differences between the PBL cases and clinical practice, however, perceptions of the reasons for the differences varied between home and host students. We are interested in the way in which this perception of difference was reported as either a stimulus or a barrier to learning.

## Introduction

Patients, medical students and doctors have been crossing national boundaries for some time; now medical curricula are on the move as the medical education sector internationalises ( Knight, 2006). Cross-border curriculum partnerships (CCPs), where an institution in one country hosts a medical curriculum developed primarily in another country, are becoming more commonplace ( Waterval, 2015). CCPs strive to provide equivalent curricula and learning experiences to students in geographically separate locations.

In 2011, St George’s University of London UK (SGUL, home institution) collaborated with the University of Nicosia (UNic, host institution), to deliver its established four-year, graduate, MBBS programme in Nicosia, Cyprus. Like all medical CCPs, the partnership faced challenges in meeting dual regulatory requirements, negotiating differences in health care practice, health care services, educational resources and educational culture between the two sites ( Waterval, 2014). These challenges were actively addressed by developing strong interactions between counterparts at every level of the partnership.

As SGUL shares its site with St Georges Hospital NHS Trust, a busy frontline hospital for a diverse local community and a tertiary centre for specialist conditions, the health services context and clinical relevance of the curriculum is particularly well developed in the early years. Clinically orientated problem-based learning cases (PBL) drive each learning week in Year 1 and 2, with associated patient contact in clinical and community services. In this way PBL provides the cognitive dimension and clinical visits the commitment dimension of Koens model (2006) for enriched learning in context. References to the configuration of UK health services are deeply embedded throughout the curriculum, and provide the backdrop for all clinical activity and decision making in PBL cases.

As health services differed between SGUL and UNic, early partnership efforts were directed towards adapting PBL cases to the local configuration of health services. Community and clinical visits provided diagnostically similar patient contact within the local service structure. However, the Cypriot regulatory body, the Tertiary Education Board, required that all teaching materials such as PBLs and lectures should be the same as the UK programme. Consequently, UNic students study PBL cases with a UK service backdrop and receive additional resources to support their experience in local health services (Nicolaou, 2014).

While this particular context is case-specific, unless CCPs develop curricula that are truly international in design, rather than imported ( Harden, 2006), the additional learning faced by host students about the home health system is a common challenge. Our study explored attitudes and learning behaviours in the two cohorts related to learning about the home system.

Given the educational mission of CCPs, host institutions with CCPs attract a more heterogenous group of students in terms of country of origin. We hypothesised that host students in our CCP would be more conscious and more engaged with understanding multiple health systems than home students. To explore how host students met their learning needs with regard to the home health system, we then sought to compare the learning behaviours for students in both sites to meet their curricular objectives.

## Methods

The study was a questionnaire based investigation of Year 1 MBBS students in SGUL and UNic investigating perceptions and experiences related to learning about health services in the curriculum. The questionnaire comprised closed and open questions (Supplementary File 1).

### Study Population

All year 1 students on the graduate MBBS at SGUL and UNic were invited to participate in this study. Participation was voluntary and responses were kept confidential. The year 1 cohort was most suitable as the curriculum consists of weekly PBL cases with community and clinical visits linked to the case of the week. Year 2 PBL case have a greater acute hospital focus and felt to be less illustrative of the range of the UK health service system.

### Questionnaire

Given the lack of validated instruments in this area, the survey was exploratory in intention. A questionnaire was developed by a small group which included a learning developer, a research assistant experienced in questionnaire design and two clinical academics. One academic had a background in optometry (the specific PBL under scrutiny) and and the other had expertise in PBL curriculum design and cross-border MBBS programme development. Questionnaire items were designed to tap into five areas of comparison between the cohorts were identified by the development group, influenced by the specific context of the CCP under study and PBL group process ( Schmidt, 2011).

### The areas of comparison were

• The heterogeneity of each cohort in terms of exposure to health systems internationally
• Whether students felt that learning about the NHS and health services worldwide was relevant to this stage in their course
• Learning behaviours when faced with health services learning issues
• Sources of learning about health services in the curriculum
• The impact discrepancies between health services in PBL and clinical experience had on learning

The first two items related to cohort comparison in relation to health systems interest and exposure. It was important not to make assumptions about students previous knowledge of and exposure to health systems. The remaining three explored the dynamics of learning from learning issue generation in PBL, through sourcing information, to evaluating impact. The generation of learning issues is key to the PBL process and the investigation therefore needed to explore what happens when students encounter a gap in their knowledge. Furthermore, we were interested in the relationships between ways in which students ascribe meaning to the gaps in the knowledge, where they perceive they originate from and how they address the gap. In order to provide a specific context for student reflection about PBL group process, some questions were directed at the health service context in a specific optometry PBL case.

The questionnaire consisted of twelve items and demographic information. These questions were in the style of 5 point Likert scale questions to gauge how much students felt they knew, or how much they agreed with statements, yes/no closed questions to ask about specific occurrences and free text questions to enable students to expand on their yes/no responses.

The questionnaire was administered on 30 May 2014 to the Year 1 SGUL students and 5 June 2014 in UNic. This was four and eight days after the PBL case respectively. The questionnaire was distributed after a lecture and also by email to the whole year to capture any students who had not attended the lecture and wished to participate in the survey. Non-responders did not differ from responders in terms of demographic characteristics.

### Ethics

Approval for the project was obtained from St George’s, University of London Ethics Review Board. In UNic, approval was obtained from the Cyprus National Bioethics Committee. Each student participant was given a written explanation of the survey and written consent was obtained.

### Survey analysis

In SGUL, out of a possible 111 students, 90 questionnaires were completed and used (81%). In UNic, out of a possible 90 students, 71 questionnaires were completed and used (78.9%), another 8 were completed but the consent form was not signed and so were not used.

Quantitative questionnaire data was analysed using IBM SPSS and summarised as frequencies and percentages for descriptive purposes. Fisher’s exact test was used to analyse the ways in which the variables were associated. Content analysis was undertaken to examine the qualitative data gained from the open text question using an editing approach (Robson, 2002). This analysis was undertaken independently by three members of the research team, two at SGUL and one at UNic, each of whom identified key themes. Final categories were arrived at by consensus-building via discussion of the considerable overlaps in individuals’ theming.

## Results/Analysis

The cohorts were similar in age and gender distribution. The mean age was 26.8 ± 5.2 (SGUL) and 24.6 ± 3.2 (UNic) with similar numbers of male and female students within each cohort. However, the UNic cohort had more diverse exposure to health services, including the UK (22.4%), USA (27.6%), Canada (22.4%) and Lebanon (10.3%) compared with UK students (9.5% with the main exposure other than UK). Notably, few UNic students had had their main exposure to health services in their country of study (5.2%). Individual students at UNic also brought experience of health services from a wide range of other countries to the programme.

With regard to perception of relevance, more of the SGUL cohort responded that learning about UK health services in the early years was important or very important, compared to the UNic cohort (SGUL 81%, UNic 66% *p=0.001*). The opposite was observed when students were asked to rate the relevance of understanding healthcare services in other countries (SGUL 20.9 %, UNic 49.3% *p=0.001*).

Based on the finding that group collaboration and individual knowledge acquisition are important for learning in PBL( Schmidt, 2011), students were asked about how often UK services generate both group and individual learning issues during PBL tutorials. The results indicate that the majority of students in both cohorts generate learning outcomes relating to UK services at least occasionally. The difference between cohorts did not reach significance (p=0.056). When asked specifically about the ophthalmology case, there was no significant difference between the number of students in each cohort who had noticed the reference to UK services (UNic 72%, SGUL 56.7% *p=0.066*). However, once noticed, it triggered a learning issue for more UNic students groups (UNic 48.4%, SGUL 36.7%, *p=0.016*).

UNic students responded that they generated individual learning issues about UK health services from PBL tutorials occasionally, often or very often more frequently than SGUL students (UNic 71.9%, SGUL 47.7%, *p= 0.005).* This was corroborated by the specific response to the ophthalmology case where Free text responses to the specific inquiry regarding the ophthalmology case demonstrated that for both cohorts the most frequent group learning objectives generated were part of the formal stated curriculum.

Faced with a learning issue, students at SGUL perceived clinical placements, being a patient and previous work experience as more important sources of learning while students in UNic received most of their information about UK services from PBL and lectures (p<0.01).

**Figure 1.**
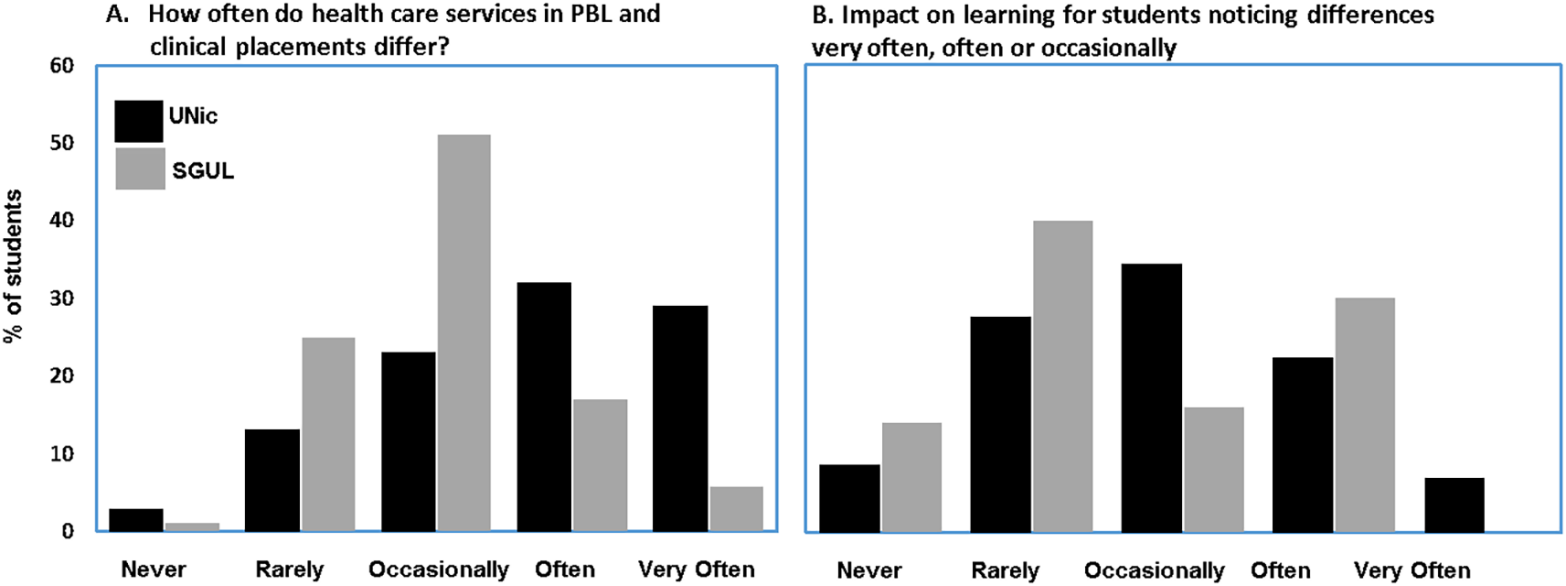
Differences between health care services in PBL and clinical placements.

Figure 1 summarises results for how often students noticed differences between UK services in PBL and their clinical placement experience as well as how often this impacted on their learning. More students studying in UNic found differences often or very often as compared to SGUL (UNic 60.9% SGUL 22.7% p<0.001). This might be expected but, given the heterogeneity of the UNic cohort, and to a lesser extent, the SGUL cohort, needed to be tested. Despite this, when differences were observed, there was no significant difference how often it impacted on their learning (impact on learning often or very often UNic 29.3% SGUL 30% *p=0.046).*

Students were invited to offer open text describing their experience of discrepancies between PBL case material and clinical visits. This provided us with an interesting “live” account of the challenge in their eyes. Analysis was concerned with explanations that students ascribed to differences and the impact on learning.

Where differences between health care services in PBL and clinical placements were noted, SGUL students tended to explain this as either natural diversity in practice (3/13 comments), a theory-practice gap caused by resource constraints in the NHS (5/13) or out of date information in PBL cases (5/13). In contrast, all comments from UNic students ascribed discrepancies to differences either between clinical guidelines or health services between the UK and Cyprus (8/8).

Comments describing the impact on learning of differences can be arranged along a continuum from a positive stimulus leading to understanding diversity of practice, through difference as a problem that can be pragmatically viewed and overcome, to difference as a negative barrier to learning.

A minority of students at both sites viewed differences as a positive stimulus (3/14 SGUL, 2/18 UNic):

“Really it just makes me look into it more and highlights the grey areas in medicine” (SGUL)

“The few times that it affects my learning it was actually a minor point and ultimately it helps me appreciate the differences in healthcare services in different countries.”(UNic)

A significant number at UNic viewed differences pragmatically as an inevitable part of a crossborder programme (7/18 UNic,)

“I need to ensure I understand NHS guidelines” (UNic)

“I have to learn multiple systems”(UNic)

Half the cohort at UNic (9/18) and the majority of the cohort at SGUL (10/14) described a negative impact on learning.

“It’s frustrating when they don’t match up and we don’t know what is the ‘right’ way” (SGUL)

“If things are not consistent, then we don’t know what we are supposed to know” (UNic)

However, some comments show that the student is in the process of moving along the continuum, sometimes painfully, from a view of medicine as a set of fixed and correct practices to a more nuanced view of a set of possible practices

“It just confuses me. I don’t know which is the right way but it also shows that each has his own way and makes us aware of different ways in healthcare systems”. (UNic)

## Discussion

To our knowledge this exploratory study comparing the perceptions and experiences of students with regard to learning about health services in a crossborder medical education partnership is the first of its kind reported in the literature.

Some of the findings, such as the more diverse exposure to worldwide health services in the UNic cohort was as we expected. UNic student groups draw upon a greater range of international health services experiences to inform their learning and we conclude that this either leads to more interest in international health services provision, or students with this orientation are more likely to choose to learn on a crossborder curriculum partnership.

Learning about health services is an active part of the PBL tutorial at both SGUL and UNic. Faced with needing to know about UK services, UNic students mainly use the PBL case and lectures to elaborate their knowledge. Many clinical lecturers were consultants in the NHS prior to teaching on the UNic programme, and can support students in their understanding. In contrast, UK students tend to use direct experience from being a patient, work experience or clinical placements to elaborate their understanding.

Student groups in both cohorts generate PBL learning issues about health services that are part of the formal learning requirements of the week, and therefore are likely to be assessed. Assessment drives learning in the sense that students are likely to devote more attention to course material they know will be assessed ( Wormald *et al*., 2009) and so we would expect students to pay attention to material that relates to their learning requirements. While both cohorts report being active about learning about health services in PBL, the specific example demonstrated that UNic student groups and individuals are working harder in PBL to meet the curricular requirements around these topics. This supports the observation that students in the host country have a greater burden of learning and work harder to meet curricular outcomes (2014 General Medical Council).

Of greater interest, however, is how students in both cohorts construct mental models to explain, interrogate and resolve differences between health services descriptions in PBLs and that experienced in clinical placements. While this was a more frequent occurrence at UNic, 22.7% of SGUL students still notice differences often or very often.

Most of the time, students do not report that differences impact on their learning. However when it does, the reality is that only a minority of students in both sites find differences a positive stimulus for learning, encouraging higher order thinking and situational interest. This may relate to the intrinsic cognitive load already being high on the medical undergraduate curriculum (Merrienboer, 2010), leading to health service differences being perceived as an extraneous load; a challenge without opportunity. There is a greater pragmatism in UNic about the situation, despite the more limited opportunities for elaborating their knowledge of UK services open to them.

## Conclusion

Variations in health services in different countries are seen both as a key challenge in medical CCPs ( Waterval, 2015) and an opportunity for the professional development of students ( Leask, 2003). While there is the burden of dual curriculum requirements, internationalisation can prepare students for professional participation across national and cultural boundaries ( Grant, 2013; Reid, 2004). The aspiration is that CCPs will educate a cadre of reflective practitioners that both accept that medicine is practiced a certain way in any one national context, but also bring creative thinking with a broader range of approaches for quality improvement of health services. This would require students to manage differing mental models of health services and employ higher order thinking when thinking about “how to get things done” in a particular health service context.

Early evaluation of the model used within this partnership for learning about health services demonstrates that most of the time the students are pragmatic, get on with learning, and are orientated to curricular outcomes. However, in terms of higher order aspirations for CCPs, only a minority of UNic students perceive differences as opportunities for learning. Personal communication from other CCP course directors also suggest that the enthusiasm of curriculum developers in CCPs are not always shared by the students ( McCrorie, 2015; Polack, 2016)! More surprisingly, because health services are not uniform within the UK, findings from the qualitative data suggest that challenges around health services are shared by students on the home and host programme.

We propose that, just as with constructing mental models of clinical problems ( Schmidt, 2011), students form mental models of health services. Health services provide a context and determine particular solutions to a clinical problem. Group discussion stimulated by PBL tutors and formal teaching could provide the flexible scaffolding required by students to overcome barriers to successful learning with regard to health service context. If the aspirations of CCPs to educate practitioners who can reflect on different models of health services are to be realised, we suggest further shared CCP development and research efforts. Given the urgency of a creative response to the current economic climate in health services provision, the need to learn health service solutions from each other is more pressing than ever.

## Take Home Messages

• Home and host students share challenges when learning about health services in cross-border curriculum partnerships but diversity is given different meanings
• Only a minority of home and host students see the diversity of health services as a positive stimulus for learning
• Analysis of learning behaviours regarding curricular objectives demonstrate that assessment drives learning about health services in home and host students
• Shared practice across CCP projects is required if aspirations for reflective practitioners that can work both within and across borders is to be realised

## Notes On Contributors

Shehla Baig

Dr Baig is Senior Lecturer in Medical Education at St George’s, University of London. She is a general practitioner and an undergraduate medicine curriculum designer, with a particular interest in cross-border curriculum partnerships.

Stella A Nicolaou

Dr Nicolaou is currently an Assistant Professor at the Department of Life and Health Sciences at the University of Nicosia (UNic). She was responsible for PBL tutor training and development in St. George’s University of London Medical Programme (UNic) and now leads the implementation of PBL in her present Department.

Denise Lawrence

Denise Lawrence has a background of lecturing in clinical skills at St George’s University of London. She also has previous experience in medicine and optometry.

Janette Myers

Dr Myers is Senior Lecturer in Student Learning and Support at St George’s, University of London. She is a learning developer, responsible for designing and implementing curricular and teaching and learning strategies that enable student achievement.

Mursheda Begum

Miss Begum is a PhD student based at the Division of Cancer Studies of King’s College London. Her project aims to assess ethnicity/national origin and gender distributions of members of UK professional bodies. She was previously part of the European Commission Mapping NCD project which mapped and evaluated European research publications and their respective funding within five chronic non-communicable diseases.

## Declarations

The author has declared that there are no conflicts of interest.

## Ethics Statement

Approval for the project was obtained from St George’s, University of London Ethics Review Board. In UNic, approval was obtained from the Cyprus National Bioethics Committee. Each student participant was given a written explanation of the survey and written consent was obtained.

## External Funding

This article has not had any External Funding

## References

[ref1] Grant C. B. (2013) Losing our chains? Contexts and Ethics of University Internationalisation. London: Leadership Foundation for Higher Education. Available at: https://www.advance-he.ac.uk/knowledge-hub/losing-our-chains-contexts-and-ethics-university-internationalisation( Accessed: 03/03/20).

[ref2] Hart M. (2014) New schools and overseas programmes. GMC Education Associate Workshop, London (unpublished).

[ref3] Harden R. M. (2006) International Medical Education and Future Directions: A Global Perspective. Academic Medicine. 81(Supplement). 10.1097/01.acm.0000243411.19573.58 17086041

[ref4] Knight J. (2006) Cross-border education: Conceptual confusion and data deficits. Perspectives in Education. 24, pp.15–27. Available at: https://www.ingentaconnect.com/content/sabinet/persed/2006/00000024/00000001/art00006( Accessed: 03/03/2020).

[ref5] Koens F. Mann K. V. Custers E. J. F. M. and Cate O. T. J. T. (2005) Analysing the concept of context in medical education. Medical Education. 39(12), pp.1243–1249. 10.1111/j.1365-2929.2005.02338.x 16313584

[ref6] Leask B. (2003), Venturing into the unknown: A framework and strategies to assist international and Australian students to learn from each other.in Bond C. & Bright P. (eds.), Research and Development in Higher Education: Learning for an unknown future. Vol 26, Higher Education Research and Development Society of Australasia. Inc., Christchurch, New Zealandpp.380–387.

[ref7] McCrorie P. Nicolaou S. and Baig S. (2015) How to set up a medical school in another country-the London-Cyprus experience after four years. Glasgow, AMEE 2015, https://amee.org/getattachment/home/AMEE_2015_Glasgow_web-(2).pdf( Accessed: 03/03/2020).

[ref8] Nicolaou S.A. Baig S. and McCrorie P. (2013) Are the PBL cases from a UK-based medical school transferable to an international cohort? The London-Cyprus experience, Prague, AMEE 2013, https://amee.org/getattachment/Conferences/AMEE-Past-Conferences/AMEE-Conference-2013/AMEE-2013-ABSTRACT-BOOK-updated-190813.pdf( Accessed: 03/03/2020).

[ref9] Reid A. and Loxton J. (2004) Internationalisation as a way of thinking about curriculum development and quality.in Carmichael R. (Ed.) Quality in a Time of Change: Proceedings of the Australian Universities Quality Forum 2004 Adelaide, Australia 7-9 July 2004, pp.99–103.

[ref10] Robson C. (2011) Real world research: a resource for social scientists and practitioner-researchers. Oxford: Blackwell.

[ref11] Schmidt H. G. Rotgans J. I. and Yew E. H. (2011) The process of problem-based learning: what works and why. Medical Education. 45(8), pp.792–806. 10.1111/j.1365-2923.2011.04035.x 21752076

[ref12] Merriãnboer J. J. G. V. and Sweller J. (2010) Cognitive load theory in health professional education: design principles and strategies. Medical Education. 44(1), pp.85–93. 10.1111/j.1365-2923.2009.03498.x 20078759

[ref13] Waterval D. G. J. Frambach J. M. Driessen E. W. and Scherpbier A. J. J. A. (2014) Copy but Not Paste: A Literature Review of Crossborder Curriculum Partnerships. Journal of Studies in International Education. 19(1), pp.65–85. 10.1177/1028315314533608

[ref14] Waterval D. G. J. Frambach J. M. Pool A. O. Driessen E. W. (2015) An exploration of crossborder medical curriculum partnerships: Balancing curriculum equivalence and local adaptation. Medical Teacher.pp.1–8. 10.3109/0142159x.2015.1019439 25776229

[ref15] Wormald B. W. Schoeman S. Somasunderam A. and Penn M. (2009) Assessment drives learning: An unavoidable truth? Anatomical Sciences Education. 2(5), pp.199–204. 10.1002/ase.102 19743508

