# Supplementary material for: Student learning about UK health services in a cross-border curriculum partnership; the London-Cyprus experience
Source: MedEdPublish (2016). 2020 Mar 30;9:58. [Version 1] doi: 10.15694/mep.2020.000058.1 (PMC10697467; doi:10.15694/mep.2020.000058.1)
Supplement: Peer review history

# Reviewer report: Reviewer response for version 1

**DOI:** 10.21956/mep.19963.r30336

**Published:** 2020-04-23

**Authors:** Keith Wilson

**Affiliations:** 1 Dalhousie University

This review has been migrated. The reviewer awarded 4 stars out of 5 The authors present a well-constructed paper that highlights the differences in student perspectives of a Problem-Based Learning curriculum delivered in two different countries/contexts. The paper utilized quantitative and qualitative approaches to address the hypotheses. It was interesting to discover that students in the host/remote programme generated more learning issues about the UK health system than in the country of origin/home students. The authors highlighted that acquisition of this knowledge was different in the two cohorts: country of origin students cited clinical placements, work experience and even being a patient whereas host students garnered their knowledge from course materials. This was despite the host students having more diversity in exposure to health systems in general. Although the was data collected in 2014, the results are applicable in a contemporary context. The question the reader is left with is that only a minority of students in both sites found the differences in health services a positive stimulus for learning. This certainly would be a launching point for further exploration.This paper was a pleasure to read and can be applicable to numerous contexts, including distributed learning contexts within a country/region.

Reviewer Expertise:

NA

No decision status is available

# Reviewer report: Reviewer response for version 1

**DOI:** 10.21956/mep.19963.r30333

**Published:** 2020-04-14

**Authors:** Johnny Lyon Maris

**Affiliations:** 1 Southampton GP Education Unit

This review has been migrated. The reviewer awarded 4 stars out of 5 Thankyou for this article. It is interesting that learning from other health systems usually occurs at a senior level of post-graduate specialisation - either as a fellowship or study tour. Here we see those differences and resultant scholarship at an undergraduate level which should lead to rich learning. The most telling paragraph in the article for me was:With regard to perception of relevance, more of the SGUL cohort responded that learning about UK health services in the early years was important or very important, compared to the UNic cohort (SGUL 81%, UNic 66% p=0.001). The opposite was observed when students were asked to rate the relevance of understanding healthcare services in other countries (SGUL 20.9 %, UNic 49.3% p=0.001).Those students seemed more interested in their health service from their country of origin, and perhaps the curriculum should take note of this in international intakes in all Medical Schools. This could then be expanded to understanding of differences and acknowledgement that there are positives and negative from all health systems.

Reviewer Expertise:

NA

No decision status is available

# Reviewer report: Reviewer response for version 1

**DOI:** 10.21956/mep.19963.r30334

**Published:** 2020-03-31

**Authors:** Deb Halder

This review has been migrated. The reviewer awarded 4 stars out of 5 The paper has systematical order of construction. It follows logical order structure and arguments along with the reasonable references as proof of the statement. The best part of this paper is its detailed description of methodology. It tended to do frequency and ratio measurement as per the quality of a quantitative research.But the authors have offered analytical descriptions of responses from open ended questions which extends the thought of qualitative nature of the research. Thus, the paper has amalgamated both the quantitative and qualitative research perspectives though the authors failed to touch on this matter at the methodology section of the paper.The Conclusion has been divided with two parts with a recommendation at the later part which highlights the necessity of the research and its implication for the future arena of knowledge. But the paper could have provided the procedures how so many responses were authenticated, if there prevailed any intervening variables while acquiring responses through mail. How mail generated responses were validated could have been dealt with.

Reviewer Expertise:

NA

No decision status is available

# Reviewer report: Reviewer response for version 1

**DOI:** 10.21956/mep.19963.r30335

**Published:** 2020-03-30

**Authors:** Felix Silwimba

**Affiliations:** 1 University of Lusaka

This review has been migrated. The reviewer awarded 5 stars out of 5 this is an informative and challenging study. it is important for linking national health system and medical education requirements.

Reviewer Expertise:

NA

No decision status is available
